# SARS-CoV-2 vaccine breakthrough infection in the older adults: a meta-analysis and systematic review

**DOI:** 10.1186/s12879-023-08553-w

**Published:** 2023-09-04

**Authors:** Xiaohui Jing, Menglin Han, Xiaoxuan Wang, Li Zhou

**Affiliations:** 1 School of Chinese Materia Medica, Tianjin University of Traditional Chinese Medicine, 10 Poyang Lake Road, Tianjin, 301617 P.R. China; 2 College of Pharmaceutical Engineering of Traditional Chinese Medicine, Tianjin University of Traditional Chinese Medicine, 10 Poyang Lake Road, Tianjin, 301617 P.R. China

**Keywords:** COVID-19, Vaccine, Breakthrough infection, Older adults, Meta-analysis, Systematic review

## Abstract

**Background:**

Corona Virus Disease 2019 (COVID-19) mRNA vaccine effectiveness (VE) has recently declined, and reports about COVID-19 breakthrough infection have increased. We aimed to conduct a meta-analysis on population-based studies of the prevalence and incidence of severe acute respiratory syndrome coronavirus 2 (SARS-CoV-2) breakthrough infection amongst older adults worldwide.

**Methods:**

Studies from PubMed, Embase, Cochrane Library, and Web of Science were systematically screened to determine the prevalence and incidence of SARS-CoV-2 breakthrough infection in older adults from inception to November 2, 2022. Our meta-analysis included 30 studies, all published in English. Pooled estimates were calculated using a random-effect model through the inverse variance method. Publication bias was tested through funnel plots and Egger’s regression test, and sensitivity analyses were performed to confirm the robustness of the results. This research was performed following the Preferred Reporting Items for Systematic Reviews and Meta-Analyses (PRISMA) guidelines.

**Results:**

Thirty publications were included in this meta-analysis (17 on prevalence, 17 on incidence, and 4 on both). The pooled prevalence of COVID-19 breakthrough infection among older adults was 7.7 per 1,000 persons (95% confidence interval [95%CI] 4.0–15.0). At the same time, the pooled incidence was 29.1 per 1000 person-years (95%CI 15.2–55.7).

**Conclusions:**

This meta-analysis provides estimates of prevalence and incidence in older adults. We concluded that the prevalence and incidence of SARS-CoV-19 breakthrough infection in older people was low. The prevalence and incidence of breakthrough infection admitted to hospital, severe-critical, and deathly was significantly lower. Otherwise, there was considerable heterogeneity among estimates in this study, which should be considered when interpreting the results.

**Supplementary Information:**

The online version contains supplementary material available at 10.1186/s12879-023-08553-w.

## Introduction

The COVID-19 pandemic caused by SARS-CoV-2 continues to constitute a public health emergency of international concern (PHEIC). Moreover, COVID-19 with a high number of deaths compared to other respiratory infectious diseases [1]. Globally, as of February 22, 2023, there have been COVID-19, including 6,86,850,594 deaths and 757,264,511 confirmed cases, reported to WHO [2].

Currently, COVID-19 vaccination is recognized as one of the most extraordinary measures to control the global COVID-19 pandemic, reducing the morbidity of COVID-19. As of February 21, 2023, there were 180 candidate vaccines in clinical development and 199 in pre-clinical development, including the following main categories: protein subunit vaccines, viral vector vaccines, inactivated virus vaccines, and DNA- and mRNA-based vaccines, among others [3].

Vaccines against SARS-CoV-2 have effectively prevented COVID-19, but rare breakthrough infections have been reported. A study found that the immune effect of older adults after being vaccinated with COVID-19 vaccines was less effective than that of young people. Still, safety is higher in older people after vaccination [4]. Older adults may represent a vulnerable group with a higher risk of breakthrough infection, worse outcomes, and even death from COVID-19 than young adults. Therefore, there is a concern for the prevalence and incidence rate of SARS-CoV-2 breakthrough infection amongst older adults. Based on studies, we conducted a meta-analysis to estimate the prevalence and incidence of SARS-CoV-2 breakthrough infection amongst older adults and compare the prevalence and incidence in different degrees of severity.

We adopted the definition of breakthrough infection as testing positive for SARA-COV-2 via reverse-transcription polymerase chain reaction (RT-PCR) or rapid antigen-detection diagnostic test from any sample (i.e., nasal swab, nasal wash, nasopharyngeal swab oropharyngeal swab, saliva, sputum, bronchoalveolar lavage fluid, pleural fluid, or lung tissue.) in any clinical setting regardless of the degree of severity after 14 days since the last dose of any SARS-CoV-2 vaccine [5–7].

## Materials and methods

### Search strategy

A systematic literature search was conducted using PubMed, Cochrane Library, Embase, and Web of Science databases to identify all relevant studies until November 2, 2022. The snowballing method was used to hand searching of the reference lists of relevant studies during the same period.

The MeSH terms were:

1. “COVID-19 breakthrough infections” [Title/Abstract])
2. “elderly” [Title/Abstract]
3. “COVID 19 Vaccines” [Title/Abstract]

The above search terms were run in PubMed and were customized for each database when necessary.

The literature obtained by the systematic search is managed by NoteExpress, and duplicate literature is deleted with NoteExpress. We screened the titles and abstracts of obtained studies to delete irrelevant studies. At last, XH J and ML H independently conducted full-text screening to determine eligibility. Any discrepancies were resolved by discussion with XX W.

### Inclusion criteria

Studies were included if (1) They contain a cohort of older adults vaccinated for at least 14 days regardless of sex and geographic location, (2) The prevalence or incidence of SARS-CoV-2 breakthrough infection among older adults was reported, or it could be calculated based on available data in the article. (3) They were cross-sectional, cohort, or randomized controlled studies.

### Exclusion criteria

Studies were excluded if (1) They were reviews, letters, comments, protocols, conference abstracts, systematic reviews, meta-analyses, case reports, animal studies, preprint studies, case–control studies, or test-negative design studies, (2) They examined older adults living in institutions (i.e., living in nursing homes, long-term care facilities or sheltered) because they were more likely to have a functional impairment and low self-rated health condition [8]. (3) They did not report sufficient data and information, and efforts to contact the authors were unsuccessful. (4) Their older population sample size was less than 500. (5) They were published in different articles with duplicate participants.

### Quality assessment and data extraction

Two reviewers assessed the risk of bias in the cohort studies using the NOS (The Newcastle–Ottawa Scale), the risk of bias in the randomized controlled studies using the Cochrane Risk of Bias tool, and the risk of bias in cross-sectional studies using the JBI (Joanna Briggs Institute) Critical Appraisal Checklist for Analytical Cross-Sectional Studies. For cohort studies, A scores from 7–9, 4–6, and 1–3 were, respectively, deemed as high, moderate, and low quality. For Cross-Sectional Studies, A score from ≥ 5, 3–4, and 0–2 were, respectively, considered as high, moderate, and low-quality [9]. Two reviewers independently assessed the quality of each included study, and disagreements were resolved by consensus. Furthermore, we contacted the authors for data collection in our meta-analysis when the article did not report enough information.

We used a standardized Excel form to extract data from the included studies, including the study author, publication year, country, study type, vaccine type and dose, study period, age range, sample size, and breakthrough infection regardless of the degree of severity, breakthrough infection at distinct severity stages (Symptomatic SARS-CoV-2 breakthrough infection, breakthrough infection admitted to hospital, Severe–critical breakthrough infection, and breakthrough infection death), person-years (convert person-days into person-years according to 365 days of a year), and the number of older adults with SARS-CoV-2 breakthrough infection. And two reviewers (XH J and ML H) independently extracted these data.

### Statistical analysis

For each study included in the analyses, the prevalence was estimated in each group by dividing the number of breakthrough infection cases in older adults by the total number of older individuals receiving the vaccine. Similarly, the incidence expressed in person-years was calculated by dividing the number of older individuals who were breakthrough infected during a specified time frame by the total number of person-years vaccinated during that time.

Statistical analyses were performed using R version 4.2.2. Pooled prevalence and incidence and corresponding 95% confidence interval (CI) were obtained using the metaprop and metarate functions in the meta statistic package [10]. Using log transformation, the random-effects model was employed through the inverse variance method to pool prevalence and incidence. We use forest plots to describe statistical results.

Heterogeneity among the studies was evaluated by the I^2^ statistics, in which I^2^ values from 50–90 may represent substantial heterogeneity, and from 75% to 100 indicated considerable heterogeneity [11]. The significance of the heterogeneity test was assessed using Cochran’s Q test, in which a p-value of < 0.05 implied significant heterogeneity. A subgroup analysis was carried out based on different degrees of severity: Symptomatic SARS-CoV-2 breakthrough infection, breakthrough infection admitted to hospital, Severe–critical breakthrough infection, and breakthrough infection death. Publication bias was tested through the apparent symmetry of the funnel plot and Egger’s regression test, where a statistically significant intercept at α = 0.05 indicated evidence of funnel plot asymmetry. And leave-one-out sensitivity analyses were performed to confirm the robustness of the results.

## Results

### Literature screening process and results

**Fig. 1:**
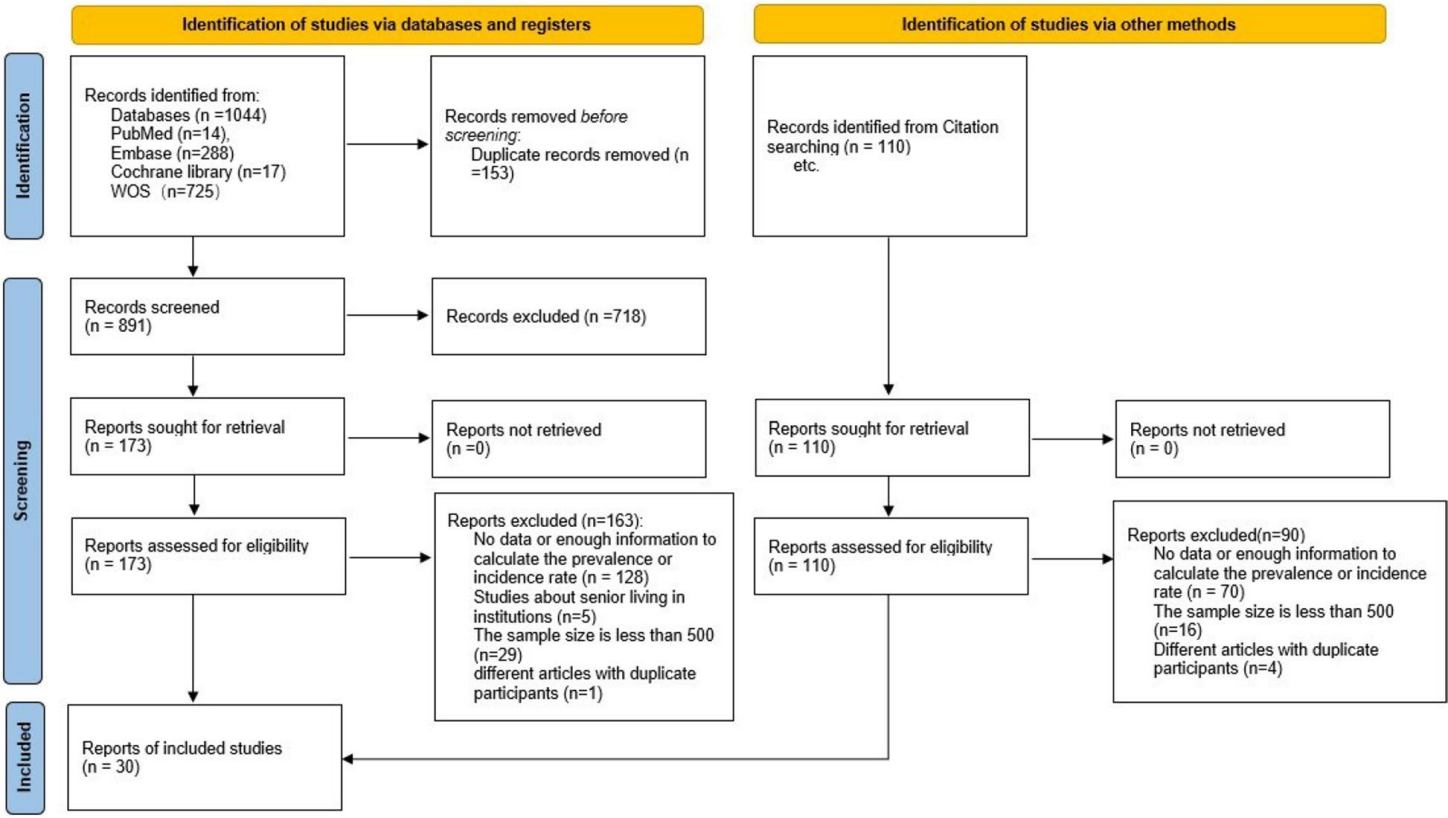
PRISMA Flow Diagram

Initially, 1,044 studies were searched from English databases, among which 14 were from PubMed, 288 from EMBASE, 17 from Cochrane Library, and 725 from Web of Science. Hand-searching contributed to 110 studies. After reading the complete text, the studies that did not meet the inclusion criteria were excluded and finally included in the meta-analysis. A total of 17 studies reported the prevalence of COVID-19 breakthrough infection among older adults, 17 on incidence, and 4 on both. The study selection procedure is outlined in Fig. 1.

### Baseline characteristics and risk of included studies

Most of the included studies were cohort studies (19/30,63.33%), cross-sectional studies (5/30, 16.67%), and randomized controlled studies (6/30,20.00%). The details of included studies are shown in Table 1. The risk of bias in included studies is graphically demonstrated in Table 2, Table 3, and Table 4. All types of studies included were of moderate to high quality. Only 4 cohort studies were of moderate quality, and the other 26 were of high quality.

**Table 1:** Characteristics of studies included in the systematic review

Study ID	Country	Study type	Vaccine type and dose	study period	age range	sample size	outcome
Barak Mizrahi 2021.11 [12]	Israel	retrospective cohort study	the BNT162b2-2dose	2021.6.1–2021.7.27	> 60	55,512	1^a^
LaithJ.Abu-Raddad 2021.11 [13]	Qatar	retrospective cohort study	the BNT162b2-2dose mRNA-1273-2dose	2020.12.21–2021.9.19	> 60	67,808	1
Aharona Glatman-Freedman 2021.10 [14]	Israel	retrospective cohort study	the BNT162b3-1 or 2dose	2020.12.21–2021.2.6	> 60	NA	12^b^3^c^4^d^5^e^
Matthew W Reynolds 2022.9 [15]	USA	Cross-Sectional study	BioNTech-2dose Moderna-2dose Ad26.COV2.S-1dose	2020.12.15–2021.9.16	> 60	2436	1
Jose-Miguel Yamal 2022.5 [16]	USA	Cross-Sectional study	BioNTech-2dose Moderna-2dose Ad26.COV2.S-1dose	2020.12.14–2021.9.30	> 60	NA^f^	1
Yinon M. Bar-ON 2022.4 [17]	Israel	retrospective cohort study	the BNT162b2-3 or 4dose	2022.1.10–2022.3.2	> 60	1,252,331	14
Sara Y Tartof 2021.10 [6]	USA	retrospective cohort study	the BNT162b2-1dose	2020.12.14–2021.8.8	> 65	8923	13
Utkarsh Agrawal 2021.12 [18]	Scotland	prospective cohort study	ChAdOx1-1dose the BNT162b3-1dose	2020.12.8–2021.4.18	> 65	912,348	3 or 5
Eli S. Rosenberg 2021.12 [19]	USA	retrospective cohort study	the BNT162b3-NA Mrna-1273-NA Ad26.COV2.S-NA	2021.5.1–2021.9.3	> 65	2,088,491	13
Ori Magen 2022.4 [20]	Israel	retrospective cohort study	the BNT162b3-3 or four dose	2022.1.3–2022.2.18	> 60	364,244	12,345
Veerle Stouten 2022.4 [21]	Belgium	Cross-Sectional study	BNT162b2 -2 dose mRNA-1273–2 dose ChAdOx1-2 dose Ad26.COV2.S-1dose	2021.2.1–2021.6.18	> 65	2,105,175	12
Amelia Green 2022.7 [22]	England	Cross-Sectional study	BNT162b2 -2dose ChAdOx1 nCoV-19-2dose mRNA-1273-2dose	2020.12–2021.11	> 60	5,583,760	1345
J. Daniel Kelly2022.9 [23]	USA	retrospective cohort study	BNT162b2 -3 dose mRNA-1273–3 dose Ad26.COV2.S-2 dose	2021.7.1–2022.5.30	> 65	1,100,248	2 3or5
Nina Breinholt Stærke 2022.8 [24]	Denmark	Cross-Sectional study	BNT162b2 -2 dose mRNA-1273-2dose ChAdOx1-1 dose	2021.7.1—2022.2.1	> 65	2918	1
Eric J Haas 2021.5 [25]	Israel	retrospective cohort study	the BNT162b3-2dose	2021.1.24–2021.4.3	> 65	1,015,620	12,345
Karin Hardt 2022.9 [26]	Belgium, Brazil Colombia France Germany the Philippines South Africa Spain the UK the USA	Randomized controlled Trail	Ad26.COV2.S -2 dose	2020.11.16–2021.6.25	> 60	1894	4
Jerald Sadoff 2022.2 [27]	Argentina Brazil, Chile Colombia, Mexico Peru South Africa The United States	Randomized controlled Trail	Ad26.COV2.S-1dose	2020.9.21—2021.7.9	> 60	6735	4
Raches Ella 2021.12 [28]	Indian	Randomized controlled Trail	BBV152-2 dose	2020.11.16–2021.5.17	> 60	893	2
Hana M El Sahly 2021.11 [29]	USA	Randomized controlled Trail	mRNA-1273-2dose	2020.10.23–2021.3.26	> 65	3626	2
scott A Halperin 2022.1 [30]	Argentina, Chile, Mexico Pakistan Russia	Randomized controlled Trail	The Ad5-nCoV vaccine-1 dose	2020.9.22.–2021.1.15	> 60	1323	24
Ann R. Falsey 2021.12 [31]	the United States Chile, Peru	Randomized controlled Trail	ChAdOx1 nCoV-19–2 dose	2020.8.28–2021.3.5	> 65	4827	2
Giulia Vivaldi 2022.11 [32]	UK	prospective cohort study	ChAdOx1 nCoV-19-2dose BNT162b2 -3 or 4dose mRNA-1273–4 dose	2020.5.1–2022.2.21	> 60	15,777	1
Alejandro Jara 2021.7 [33]	Chile	prospective cohort study	CoronaVac-2 dose	2021.2.2–2021.5.1	> 60	2,230,270	1345
Sharon Hui Xuan Tan 2022.2 [34]	Singapore	retrospective cohort study	the BNT162b2-2dose the mRNA1273-2dose	2021.9.15–2021.10.31	> 60	127,077	14
Jennifer M. Polinski 2022.3 [35]	US	cohort study	Ad26.COV2.S-1dose	2020.3.1–2021.8.31	> 65	125,402	13
Tyler N. A. Winkelman 2022.3 [36]	USA	cohort study	the BNT162b2-2dose Mrna1273-2dose Ad26.COV2.S-1dose	2020.10.25–2021.10.30	> 65	780,723	3
Massimo Fabiani 2022.2 [37]	Italy	retrospective cohort study	the BNT162b2-2dose Mrna1273-2dose	2020.12.27–2021.11.7	> 65	10,485,794	1 3or5
Julia Hippisley-Cox 2021.9 [38]	UK	prospective cohort study	the BNT162b2-1 or 2dose ChAdOx1nCoV-19–1 or 2dose	2020.12.8–2021.6.15	> 65	2,454,586	35
Peter Nordstrom 2022.3 [39]	Sweden	cohort study	the BNT162b2-2dose Mrna1273-2dose ChAdOx1nCoV-19-2dose	2021.1.12—2021.10.4	> 65	NA	1
Srinivasa Vittal Katikireddi 2022.1 [40]	Scotland	Retrospective cohort study	ChAdOx1nCoV-19-2dose	2021.1.18–2021.10.25	> 65	5,463,138	2 3or5

^a^SARS-CoV-2 breakthrough infection, regardless of the degree of severity

^b^Symptomatic SARS-CoV-2 breakthrough infection

^c^SARS-CoV-2 breakthrough infection admitted to hospital

^d^Severe–critical SARS-CoV-2 breakthrough infection

^e^SARS-CoV-2 breakthrough infection death

^f^Not available

**Table 2:** Bias assessment of the included cohort studies

Included studies	Selections				Comparability	Outcome			Total score
	Representativeness of the exposed cohort	Selection of the non-exposed cohort	Ascertainment of exposure	Demonstration that outcome of Interest was not present at start of study	Comparability of cohorts on the basis of the design or analysis	Assessment of outcome	Was follow up long enough for outcomes to occur	Adequacy of follow up of cohorts	
Barak Mizrahi-2021.11 [12]	*	*	*	*	**	*	0	*	8
LaithJ.Abu-Raddad,-2021.11 [13]	*	*	*	0	**	*	*	0	7
Aharona Glatman-Freedman-2021.10 [14]	*	*	*	*	**	*	0	0	7
Yinon M. Bar-ON-2022.4 [17]	*	*	*	*	**	*	0	*	8
Sara Y Tartof -2021.10 [6]	*	*	*	0	**	*	*	0	7
Utkarsh Agrawal-2021.12 [18]	*	*	*	0	**	*	0	0	6
Eli S. Rosenberg,-2021.12 [19]	*	*	*	*	**	*	0	0	7
Ori Magen-2022.4 [20]	*	*	0	*	**	*	0	0	6
J. Daniel Kelly,-2022.9 [23]	0	*	*	0	**	*	*	0	6
Eric J Haas-2021.5 [25]	*	*	*	*	**	*	0	0	7
Giulia Vivaldi-2022.9 [32]	*	*	0	0	**	0	*	0	5
Alejandro Jara-2021.7 [33]	*	*	*	*	**	*	0	0	7
Sharon Hui Xuan Tan 2022.2 [34]	*	*	*	*	**	*	0	0	7
Jennifer M. Polinski 2022.3 [35]	*	*	*	*	**	*	*	*	9
Tyler N. A. Winkelman 2022.3 [36]	*	*	*	*	**	*	*	*	9
Massimo Fabiani 2022.2 [37]	*	*	*	*	**	*	*	0	8
Julia Hippisley-Cox 2021.9 [38]	*	*	*	*	**	*	*	0	8
Peter Nordstrom 2022.3 [39]	*	*	*	0	**	*	*	0	7
Srinivasa Vittal Katikireddi 2022.1 [40]	*	*	*	*	**	*	*	*	9

**Table 3:** Bias assessment of the included cross-sectional studies

Included studies	Were the criteria for inclusion in the sample clearly defined?	Were the study subjects and the setting described in detail?	Was the exposure measured in a valid and reliable way?	Were objective, standard criteria used for measurement of the condition?	Were confounding factors identified?	Were strategies to deal with confounding factors stated?	Were the outcomes measured in a valid and reliable way?	Was appropriate statistical analysis used?	Total score
Matthew W Reynolds-2022.9 [15]	No	Yes	No	Yes	Yes	Yes	No	Yes	5
Jose-Miguel Yamal-2022.5 [16]	No	Yes	Yes	Yes	Yes	Yes	Yes	Yes	7
Nina Breinholt Stærke-2022.8 [24]	Yes	No	Yes	Yes	Yes	Yes	Yes	Yes	7
Amelia Green-2022.7 [22]	Yes	Yes	Yes	Yes	Yes	Yes	Yes	Yes	8
Veerle Stouten-2022.4 [21]	No	No	Yes	Yes	Yes	Yes	Yes	Yes	6

**Table 4:** Bias assessment of the included randomized controlled studies

Included studies	Random allocation	Allocation concealment	Evaluation of blindness	Data integrity	Selective report	others
Jerald Sadoff-2022.2 [27]	Low risk	Low risk	Low risk	Low risk	Low risk	Low risk
Raches Ella-2021.12 [28]	Low risk	Low risk	Low risk	Low risk	Low risk	Low risk
Hana M El Sahly-2021.11 [29]	Low risk	Low risk	Low risk	High risk	Low risk	Low risk
Scott A Halperin -2022.1 [30]	Low risk	Low risk	Low risk	Low risk	Low risk	Low risk
Ann R. Falsey-2021.12 [31]	Low risk	Low risk	Low risk	Low risk	Low risk	High risk
Karin Hardt-2022.9 [26]	Low risk	Low risk	Low risk	Low risk	Low risk	Unclear

### The prevalence of meta-analysis of outcome

#### The prevalence of breakthrough infection among older adults

**Fig. 2:**
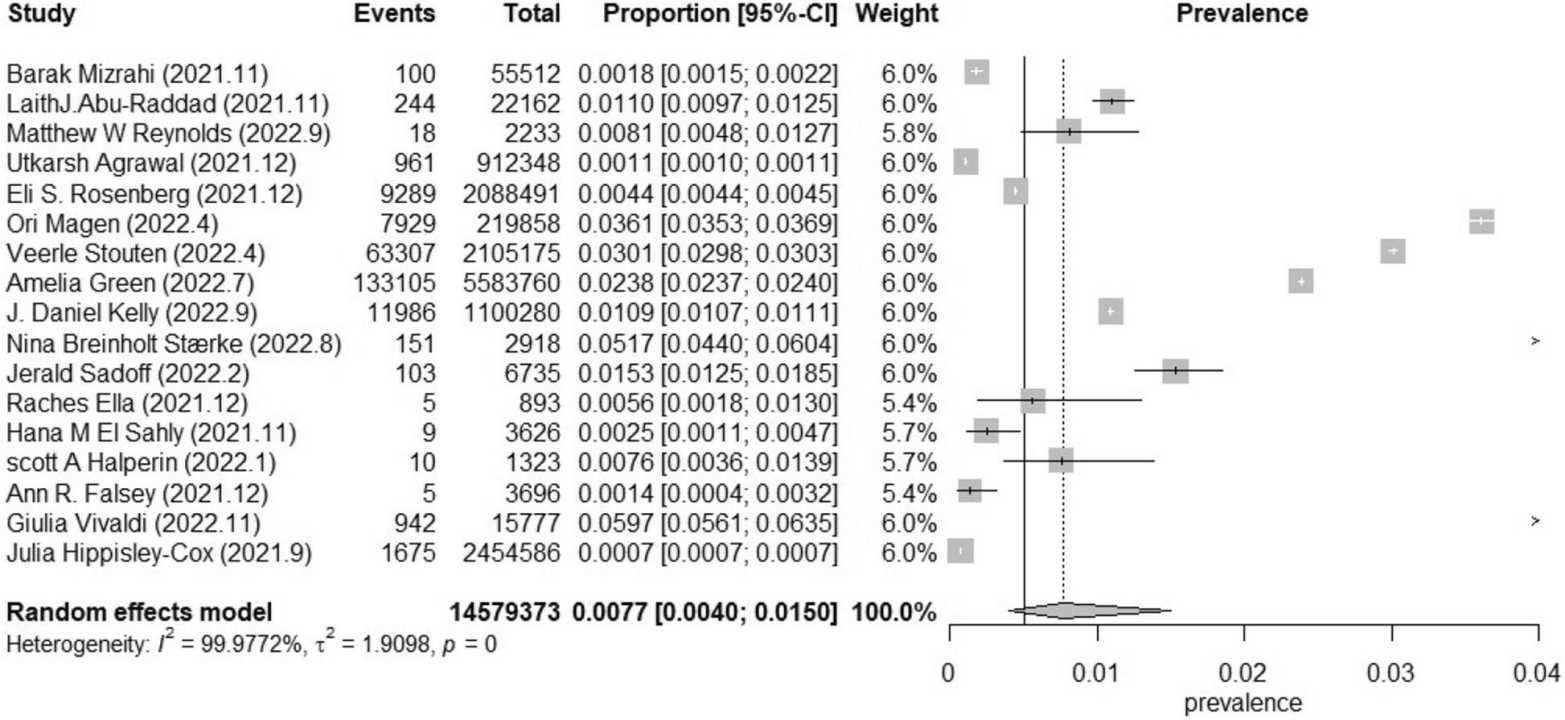
Forest plot of the pooled prevalence in older adults (Considerable heterogeneity: I2 = 99.98% and a statistically significant Q test (*p* = 0))

Based on 17 studies involving 14,579,373 older adults with different vaccines, doses, and health conditions, the prevalence ranged from 0.7 to 59.7 per 1000 individuals. The pooled prevalence of SARS-CoV-2 breakthrough infection in older adults was 7.7 (95%CI 4.0–15.0) per 1000 individuals. The studies included in this meta-analysis had considerable heterogeneity, as shown by an I^2^ value of 99.98% and a statistically significant Q test (*p* = 0) (Fig. 2).

#### Subgroup analysis based on the degree of severity of the breakthrough infection

**Fig. 3:**
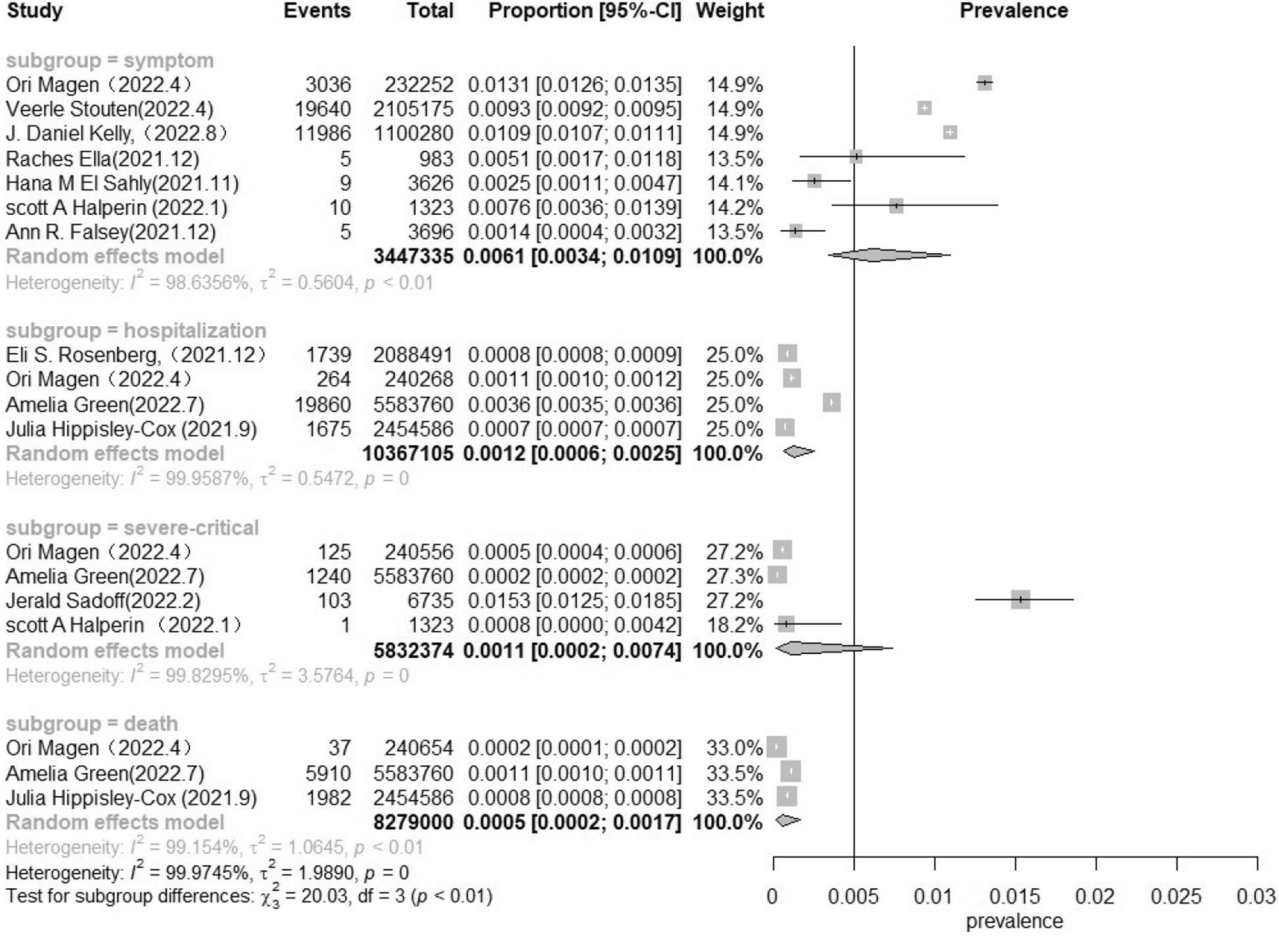
Forest plot of the pooled prevalence in older adults in different degrees of severity

Upon classifying the degree of severity of the breakthrough infection among older adults, it was noted that symptomatic breakthrough infection had a prevalence of 6.1 (3.4–10.9) per 1000 individuals, the pooled prevalence of breakthrough infection admitted to hospital was 1.2 (0.6–2.5) per 1000 individuals, the pooled prevalence of severe–critical breakthrough infection was 1.1 (0.2–7.4) per 1000 individuals, and the pooled prevalence of breakthrough infection deathly was 0.5 (0.2–1.7) per 1000 individuals (Fig. 3). By subgroup analysis of the prevalence of breakthrough infection in different degrees of severity, I^2^ was not significantly reduced.

#### Publication bias and sensitivity analysis

**Fig. 4:**
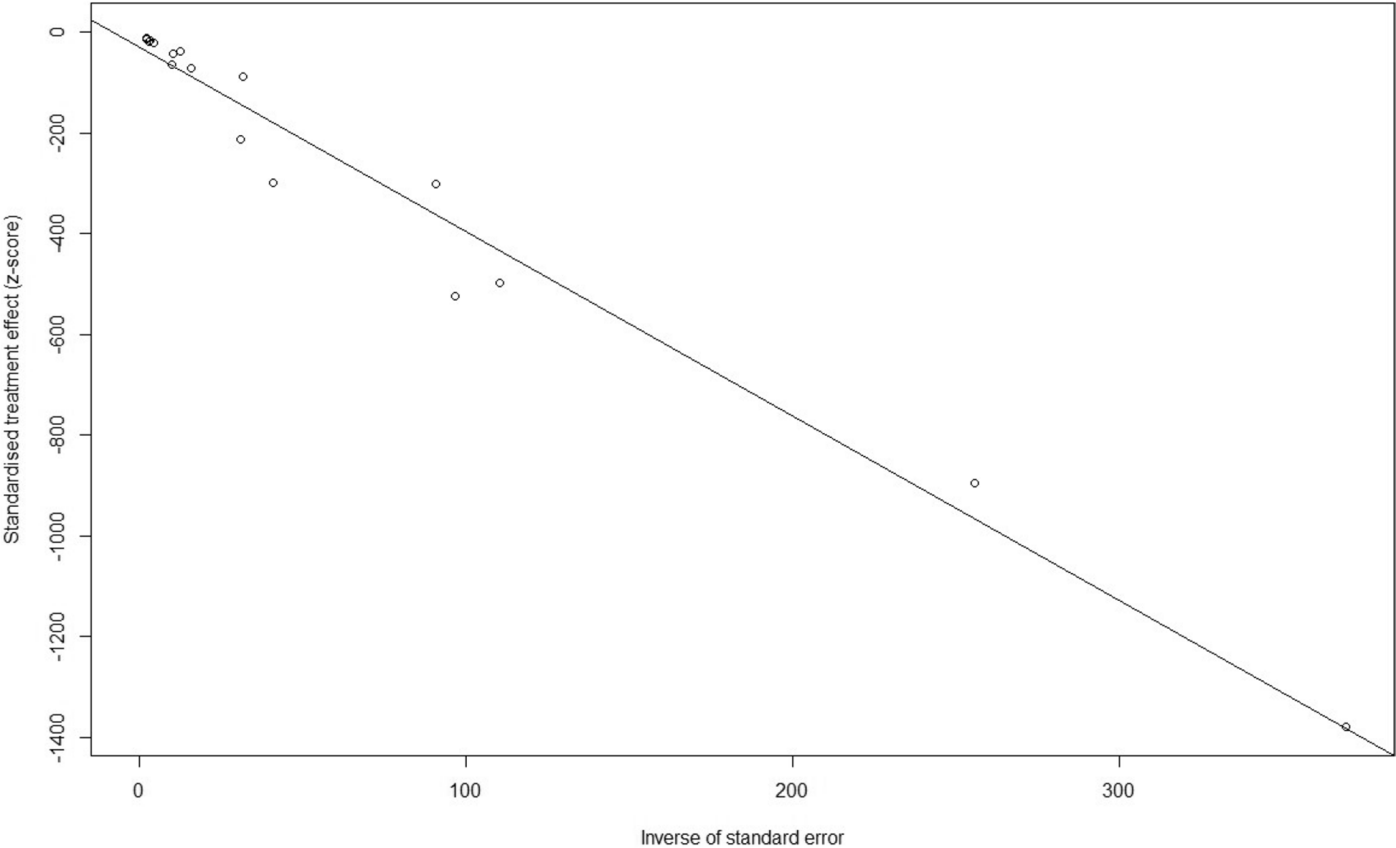
Egger test of publication bias of the included studies on prevalence

**Fig. 5:**
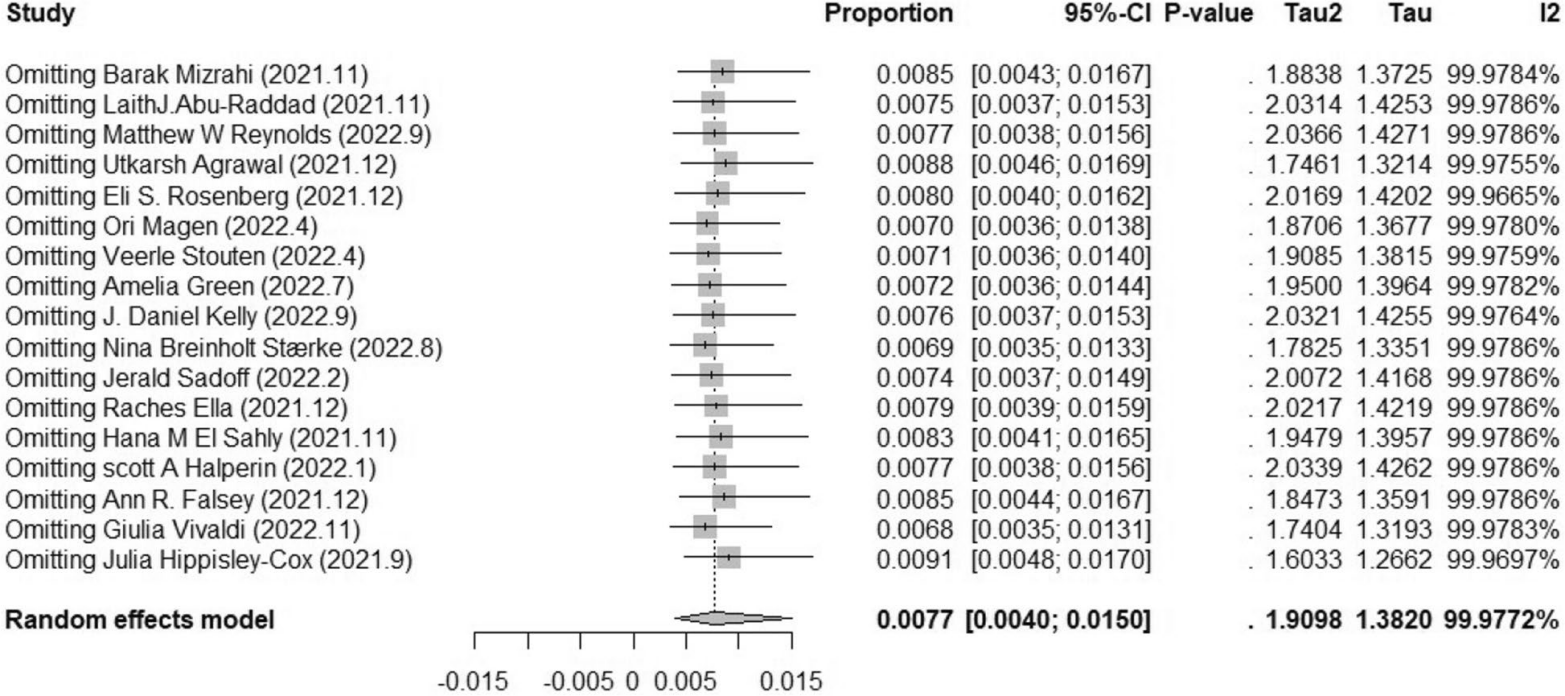
Sensitivity analysis of the included studies on prevalence

We tested the 17 studies for publication bias, and there was no evidence of publication bias for any of the estimates of prevalence using visual inspection of funnel plots or the Begg or Egger test (all *p* > 0.05) (Fig. 4). A sensitivity analysis that omitted one study did not significantly change these estimates. (6.8 per 1000 individuals versus 9.1 per 1000 individuals) (Fig. 5).

### The incidence of meta-analysis of the outcome

#### The incidence of breakthrough infection among older adults

**Fig. 6:**
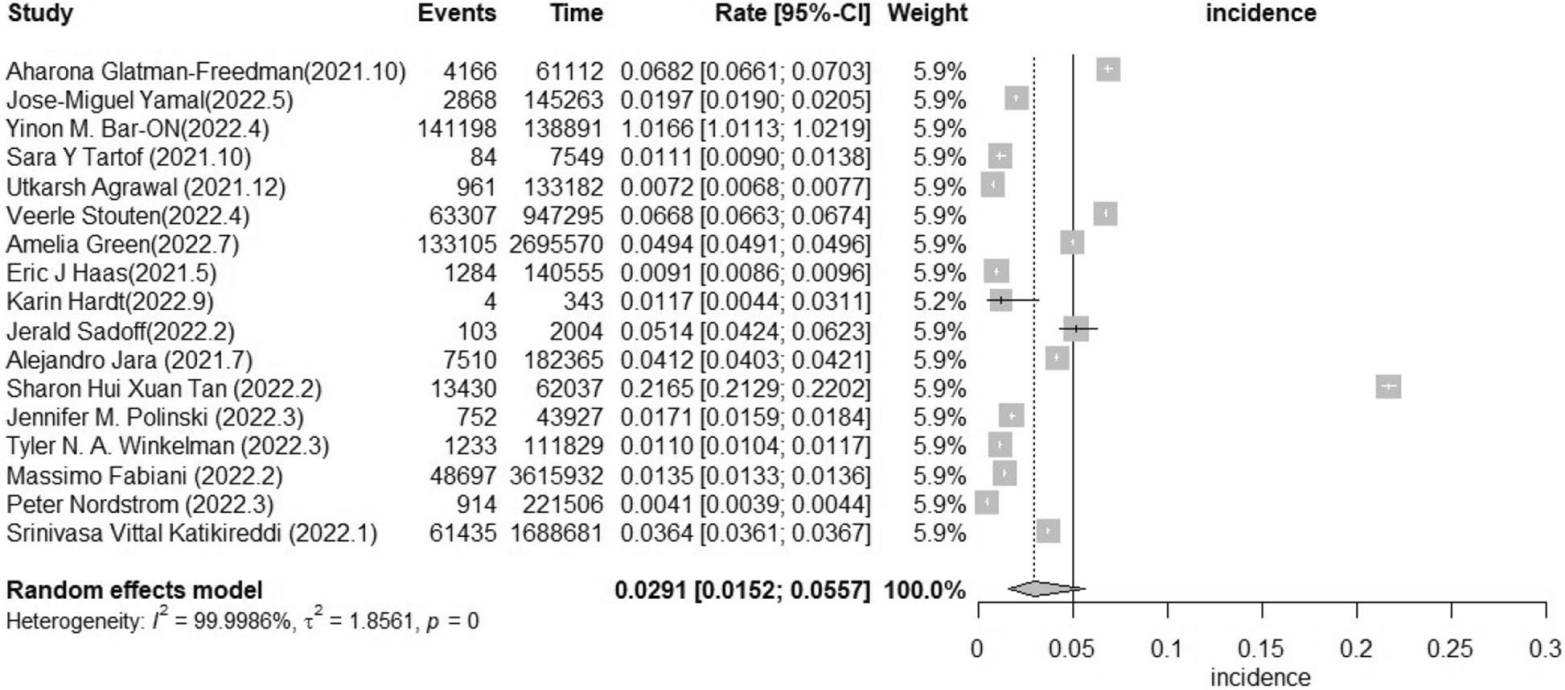
Forest plot of the pooled incidence in older adults (considerable heterogeneity: I2 = 100.00% and a statistically significant Q test (*p* = 0))

Based on 17 studies, incidence ranged from 4.1 to 1,016.6 per 1000 person-years. The pooled incidence of breakthrough infection in older adults with different vaccines, doses, and health conditions was 29.1 (15.2–55.7) per 1000 person-years. The studies included in this meta-analysis had considerable heterogeneity, as shown by an I^2^ value of 100.00% and a statistically significant Q test (*p* = 0) (Fig. 6).

#### Subgroup analysis based on the degree of severity of the breakthrough infection

**Fig. 7:**
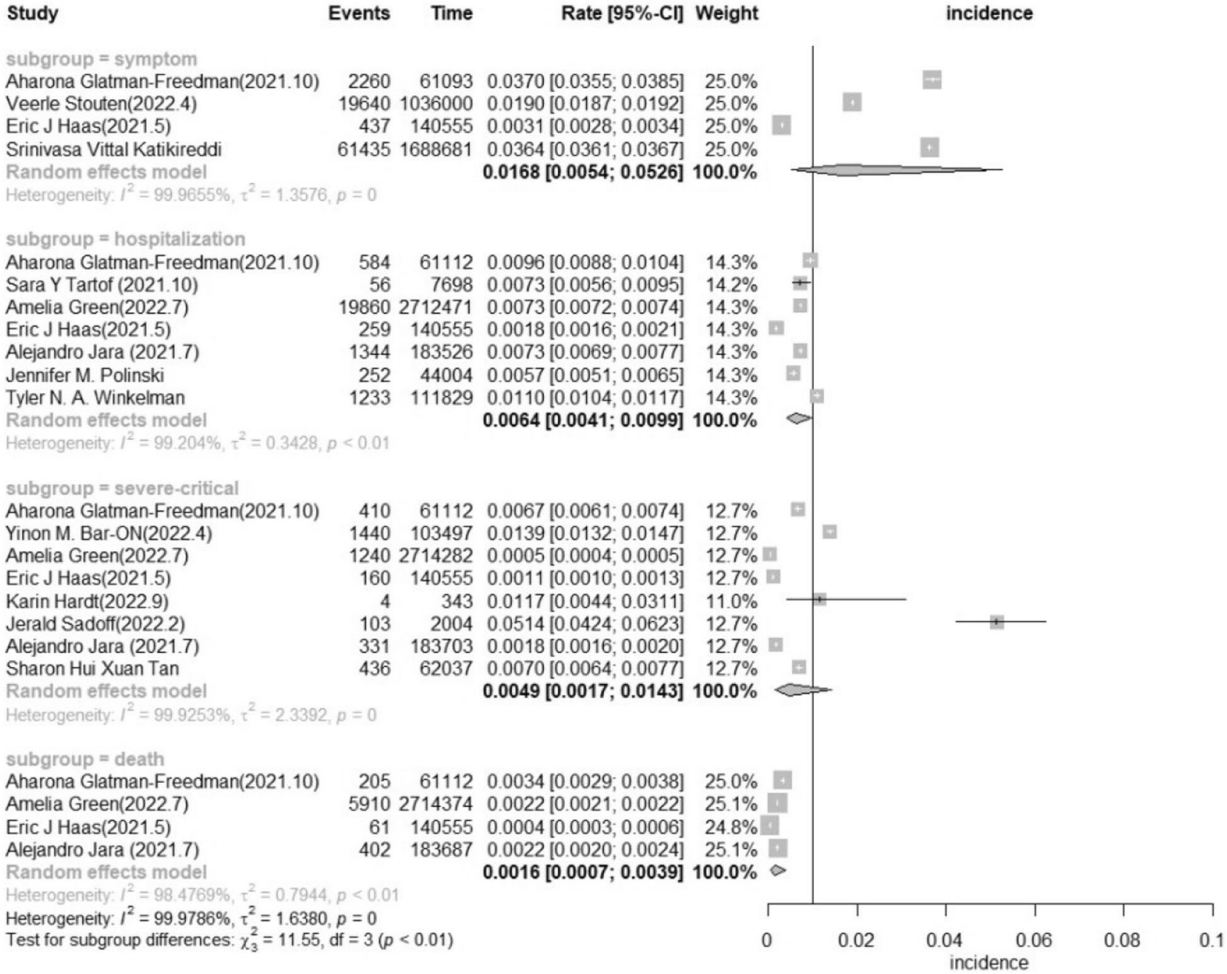
Forest plot of the pooled incidence in older adults in different degrees of severity

Upon classifying the severity of the breakthrough infection among older adults, it was noted that symptomatic breakthrough infection had an incidence of 16.8 (5.4–52.6) per 1000 person-years, the pooled incidence of breakthrough infection admitted to hospital was 6.4 (4.1–9.9) per 1000 person-years, the pooled incidence of Severe–critical breakthrough infection was 4.9 (1.7–14.3) per 1000 person-years, and the pooled incidence of breakthrough infection deathly was 1.6 (0.7–3.9) per 1000 person-years (Fig. 7). By subgroup analysis of the incidence of breakthrough infection in different degrees of severity, I^2^ was not significantly reduced.

#### Publication bias and sensitivity analysis

**Fig. 8:**
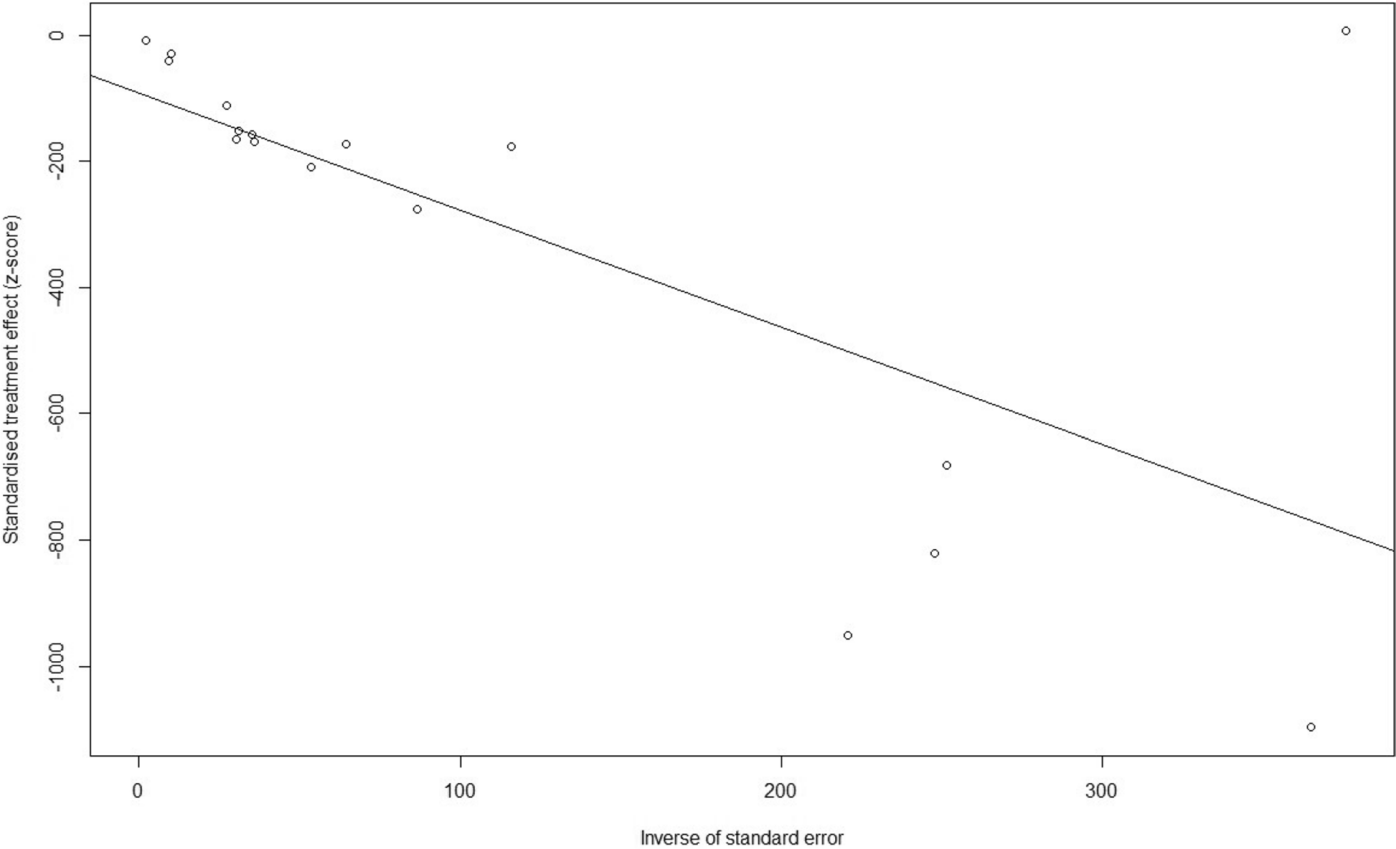
Egger test of publication bias of the included studies on incidence

**Fig. 9:**
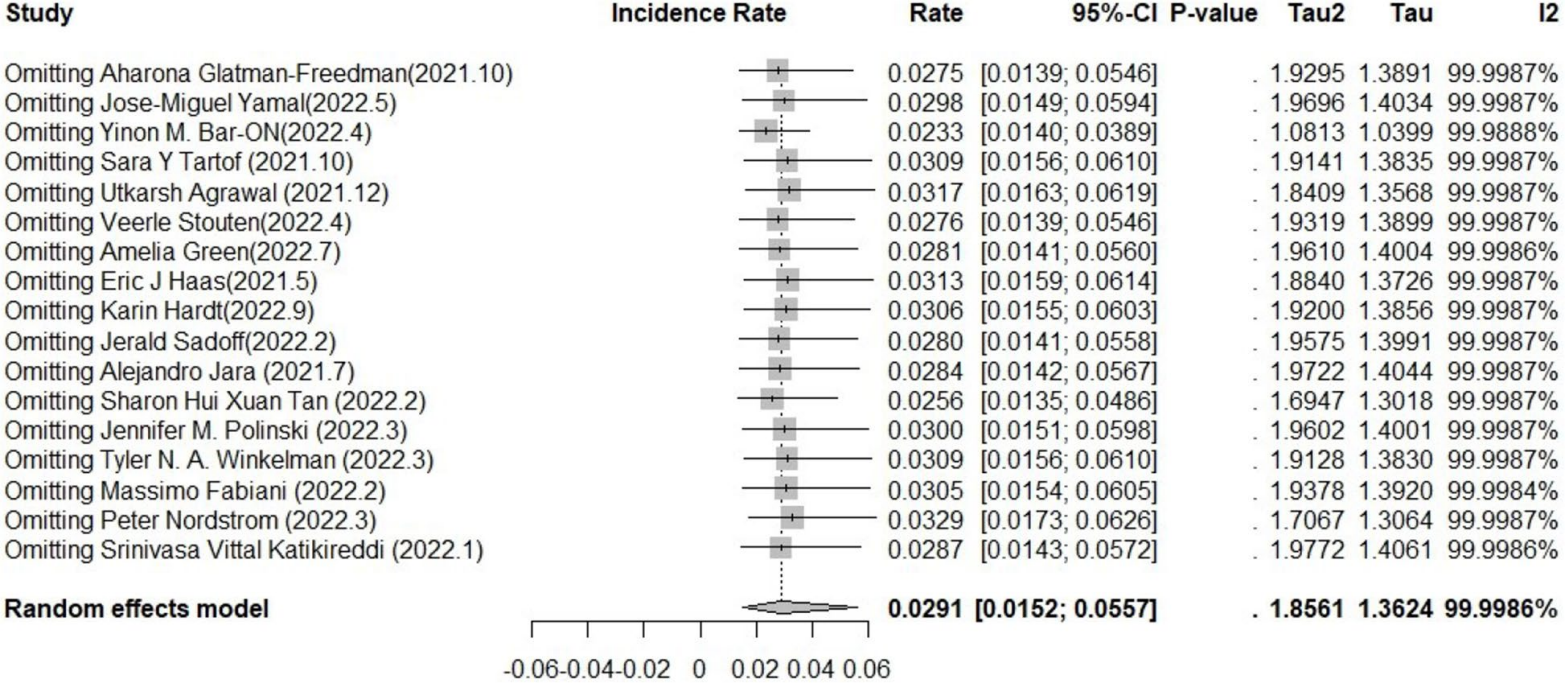
Sensitivity analysis of the included studies on incidence

We tested the 17 studies for publication bias, and there was no evidence of publication bias for any of the estimates of incidence using visual inspection of funnel plots or the Begg or Egger test (all *p* > 0.05) (Fig. 8). A sensitivity analysis that omitted one study did not significantly change these estimates. (23.3 per 1000 person-years versus 32.9 per 1000 person-years) (Fig. 9).

## Discussion

This is a meta-analysis of international studies on the prevalence and incidence of SARS-CoV-2 breakthrough infection among older adults. Our findings suggest that the pooled prevalence of breakthrough infections was 7.7 per 1000 individuals, and the pooled incidence of breakthrough infection was 29.1 per 1000 person-years. This indicates that the vaccine efficacy in older adults is still relatively high. Prevalence and incidence and corresponding 95% confidence interval (CI) were summarized using metaprop and metarate in the meta-analysis statistics package. Publication bias was tested through funnel plots and Egger’s regression test, and leave-one-out sensitivity analyses were performed to confirm the robustness of the results.

In this meta-analysis, incidence is higher than prevalence, Firstly most of the studies included in this study were observed within six months of vaccination. And different studies included were observed for different lengths, which may result in higher incidence than prevalence. In particular, people with breakthrough infections have milder symptoms, faster recovery, shorter hospital stays, and are less likely to be infectious than primary infections which may lead to a decrease in the prevalence [41–43].

We can observe considerable heterogeneity in the included studies, as indicated by high I^2^. However, this observation is common in meta-analyses estimating prevalence and incidence. Due to the nature of non-comparative proportional data, we often observe diverse point estimates among different studies than for comparatives [44]. Also, including studies with substantial sample sizes and, thus, minor standard errors led to an overestimation of the I^2^ statistic [45, 46].

Vaccines cannot provide 100% protection. Recently, estimates of COVID-19 mRNA vaccine effectiveness (VE) have declined, and reports about COVID-19 breakthrough infection have increased. The body’s immune system can remember past infections but cannot guarantee a lasting response. Some infections and immunizations provide lifelong protection, but for others, regular reminders in the form of booster shots or new, reformulated vaccines are required [47].

Immunosenescence (a process of dysfunction) occurs with age leading to changes in the immune function of older adults [48]. Combined with decreased organ spirometry and comorbidities, older adults are more likely to develop breakthrough infections. A study about hospitalized patients with breakthrough COVID-19 infection shows that the hospitalized patients with breakthrough infection were composed mainly of older men with multiple comorbidities [49]. The mortality of patients over 65 years old hospitalized with COVID-19 after a vaccine breakthrough infection remained high but might be lower than that of unvaccinated hospitalized older patients [50].

And due to the weakening of vaccine-induced immunity over time, Vaccine protection against symptomatic disease begins to decline around 10 weeks from the second dose. The waning of protection against hospitalization was more significant among older adults [51]. But the third ‘booster’ dose seems to help. One report found about 60–70% protection from an infection two weeks after the third dose, and protection from severe illness seems strong [52], also because of the increased immune evasion by SARS-CoV-2 variants. In 2022, Omicron swept the globe. Some data suggest that existing vaccines designed for the original SARS-CoV-2 cannot provide much protection against infection with the variant, even though they seem to reduce the risk of hospitalization or death [47].

Older adults with COVID-19 breakthrough infections have a lower viral load, shorter hospital stays, and are less likely to be infectious than primary infections [43]. While some of these breakthrough infections could lead to secondary transmissions, and indeed some of them did have high viral loads, the risk of onward transmission is reduced compared to primary infections. The mRNA vaccines seem to protect not only against the acquisition of infection but also against transmission of infection. We must scale up vaccination globally to robustly control infection transmission and the extent of the pandemic [51]. Therefore, vaccination is recommended for unvaccinated older people.

Vaccine allocation is also a problem in the initial rollout of vaccines when doses are still scarce. Guidelines for vaccine prioritization have been elaborated by the Strategic Advisory Group of Experts of the WHO (WHO, 2020) and the US Advisory Committee on Immunization Practices (ACIP) of the CDC (Dooling et al., 2021), making older adults one of the priority groups to be vaccinated [53].

With the emergence of new variants and the gradual increase in breakthrough infections, many countries have implemented booster dose schedules. However, in the world, only 34.4% of people received a booster by Feb 26, 2023. In some low- and middle-income countries such as India, the percentage of people who received a booster has been only 6.52% [54]. Therefore, there is an urgent need to thoroughly learn more about the safety and efficacy of boosters to reduce people's hesitancy about vaccines, and to date, there has been no significant difference in the incidence of adverse effects between the second dose and the booster dose in most clinical studies [55, 56].

Due to the increasing global demand for vaccine boosters, different booster vaccination regimens are needed to increase coverage in times of vaccine shortage. However, knowledge of the safety and efficacy of homologous or heterologous booster vaccination still needs to be improved. Therefore, there is an urgent need to assess the effectiveness and safety of different booster vaccination regimens to inform the vaccine policies of countries using these vaccines [50].

In addition, another issue to highlight is the need for more equitable distribution of COVID-19 vaccines. However, while few high-income countries’ governments understand how to vaccinate their whole populations during the pandemic, most low- and middle-income countries have been trusting the COVID-19 Vaccines Global Access (COVAX) to acquire enough doses to vaccinate 20% of their population [56]. COVAX is dedicated entirely to accelerating the development and manufacture of COVID-19 vaccines and ensuring vaccines reach poor countries. And it is might urgent to rationalize vaccination policies, particularly for older adults, frail patients, and their caregivers. Public vaccine improvement efforts should decrease all characteristics of public health risk instead of favoring its business financial characteristics [57].

### Study limitations

This report has some limitations. First, it was restricted to studies published in English, excluding potentially relevant studies in other languages. Secondly, the inclusion of studies with a sample size greater than 500 may result in the loss of small eligible studies. Thirdly, considerable heterogeneity was observed in the included studies, as indicated by high I^2^. And it was not possible to explore the causes of the heterogeneity and come up with a more valid estimate. Caution should be exercised when interpreting pooled estimates of prevalence and incidence separately. Fourthly, most of the studies included in this study were observed within six months of vaccination. More research is needed to prove that vaccines protect older people against infection for longer. Finally, some studies provided little information about the potential influencing factors such as vaccine type, vaccine dose, gender, prior infection, time from vaccination to breakthrough infection, comorbidity, and lifestyle of the included older adults on the prevalence and incidence of COVID-19 breakthrough infection. It was also impossible to conduct meta-analyses among some groups due to the less information from studies assessing those factors. so more research is needed to explore these issues.

### Publication implications

This study shows that for the elderly population, the prevalence and incidence of breakthrough infection after vaccination are low. That reduces the hesitation of the elderly for vaccination and increases people’s willingness to vaccinate. It is conducive for humans to control and termination of the COVID-19 epidemic.

### Suggestions for future study

Firstly, longer studies can be conducted to determine the prevalence and incidence of breakthrough infections over a longer period after vaccination. Secondly, the difference in prevalence and incidence of breakthrough infection between previously infected and vaccinated populations can be studied. Thirdly, if more information about the health status, different variants of SARS-CoV-2, and sex of the study population is available, the impact of these factors on prevalence and incidence can be studied. Finally, the Short- or long-term health status of older adults after breakthrough infection.

## Conclusion

The findings suggest that the prevalence and incidence of SARS-CoV-19 breakthrough infection in older people was low. Therefore, older people should be less hesitant about vaccines. Since most of the current research on breakthrough infection in the older adults is within 6 months after vaccination, a longer observation study of breakthrough infection in the older adults should be conducted to provide more adequate evidence for the protection of the older adults by vaccines.

### Supplementary Information

**Additional file 1.**

## Data Availability

All data related to the present study are available in this manuscript and supplementary files.

## Abbreviations

COVID-19: Corona Virus Disease 2019
VE: Vaccine effectiveness
SARS-CoV-2: Severe acute respiratory syndrome coronavirus 2
PHEIC: Public health emergency of international concern
RT-PCR: Reverse-transcription polymerase chain reaction
NOS: The Newcastle–Ottawa Scale
JBI: Joanna Briggs Institute
ACIP: Advisory Committee on Immunization Practices
COVAX: COVID-19 Vaccines Global Access

## References

[1] Organization WH. Statement on the fourteenth meeting of the International Health Regulations (2005) Emergency Committee regarding the coronavirus disease (COVID-19) pandemic. Available from: https://www.who.int/news/item/30-01-2023-statement-on-the-fourteenth-meeting-of-the-international-health-regulations-(2005)-emergency-committee-regarding-the-coronavirus-disease-(covid-19)-pandemic. Accessed 22 Feb 2023.

[2] WHO Coronavirus (COVID-19) Dashboard. Organization WH.2023. Available from: https://covid19.who.int/. Accessed 22 Feb 2023.

[3] COVID-19 vaccine tracker and landscape. Organization WH. 2023. Available from: https://www.who.int/publications/m/item/draft-landscape-of-covid-19-candidate-vaccines. Accessed 22 Feb 2023.

[4] Wang J, Tong Y, Li D, Li J, Li Y (2021). The impact of age difference on the efficacy and safety of COVID-19 vaccines: a systematic review and meta-analysis. Front Immunol.

[5] S. CFDC. CDC Case definition. In: Public health investigations of COVID-19 vaccine breakthrough cases : case investigation protocol. Available from: https://stacks.cdc.gov/view/cdc/105213. Accessed 22 Feb 2023.

[6] Tartof SY, Slezak JM, Fischer H, Hong V, Ackerson BK, Ranasinghe ON (2021). Effectiveness of mRNA BNT162b2 COVID-19 vaccine up to 6 months in a large integrated health system in the USA: a retrospective cohort study. Lancet.

[7] Gupta N, Kaur H, Yadav PD, Mukhopadhyay L, Sahay RR, Kumar A (2021). Clinical characterization and genomic analysis of samples from COVID-19 breakthrough infections during the second wave among the various states of India. Viruses.

[8] Luppa M, Luck T, Weyerer S, König HH, Brähler E, Riedel-Heller SG (2010). Prediction of institutionalization in the elderly A systematic review. Age Ageing.

[9] Song G, Wang M, Chen B, Long G, Li H, Li R (2021). Lower urinary tract symptoms and sexual dysfunction in male: a systematic review and meta-analysis. Front Med (Lausanne).

[10] Schwarzer G. meta: An R Package for Meta-Analysis. In: R News. 2007. Available from: https://cran.rstudio.org/doc/Rnews/Rnews_2007-3.pdf#page=40. Accessed 22 Feb 2023.

[11] Deeks JJ, Higgins JPT, Altman DG (editors). Chapter 10: Analysing data and undertaking meta-analyses. In: Higgins JPT, Thomas J, Chandler J, Cumpston M, Li T, Page MJ, Welch VA (editors). Cochrane Handbook for Systematic Reviews of Interventions version 6.3 (updated February 2022). Cochrane. 2022. Available from: www.training.cochrane.org/handbook. Accessed 4 Jul 2023.

[12] Mizrahi B, Lotan R, Kalkstein N, Peretz A, Perez G, Ben-Tov A (2021). Correlation of SARS-CoV-2-breakthrough infections to time-from-vaccine. Nat Commun.

[13] Abu-Raddad LJ, Chemaitelly H, Ayoub HH, Yassine HM, Benslimane FM, Al Khatib HA (2021). Association of Prior SARS-CoV-2 infection with risk of breakthrough infection following mRNA vaccination in Qatar. JAMA-J AM MED ASSOC.

[14] Glatman-Freedman A, Bromberg M, Dichtiar R, Hershkovitz Y, Keinan-Boker L (2021). The BNT162b2 vaccine effectiveness against new COVID-19 cases and complications of breakthrough cases: A nation-wide retrospective longitudinal multiple cohort analysis using individualised data. EBioMedicine.

[15] Reynolds MW, Xie Y, Knuth KB, Mack CD, Brinkley E, Toovey S (2022). COVID-19 vaccination breakthrough infections in a real-world setting: using community reporters to evaluate vaccine effectiveness. Infect Drug Resist.

[16] Yamal J, Appana S, Wang M, Leon-Novelo L, Bakota E, Ye Y (2022). Trends and correlates of breakthrough infections with SARS-CoV-2. Front Public Health.

[17] Bar-On YM, Goldberg Y, Mandel M, Bodenheimer O, Amir O, Freedman L (2022). Protection by a fourth dose of BNT162b2 against omicron in Israel. N Engl J Med.

[18] Agrawal U, Katikireddi SV, McCowan C, Mulholland RH, Azcoaga-Lorenzo A, Amele S (2021). COVID-19 hospital admissions and deaths after BNT162b2 and ChAdOx1 nCoV-19 vaccinations in 2·57 million people in Scotland (EAVE II): a prospective cohort study. Lancet Respir Med.

[19] Rosenberg ES, Dorabawila V, Easton D, Bauer UE, Kumar J, Hoen R (2022). Covid-19 vaccine effectiveness in New York state. N Engl J Med.

[20] Magen O, Waxman JG, Makov-Assif M, Vered R, Dicker D, Hernán MA (2022). Fourth dose of BNT162b2 mRNA Covid-19 vaccine in a Nationwide setting. N Engl J Med.

[21] Stouten V, Hubin P, Haarhuis F, van Loenhout JAF, Billuart M, Brondeel R (2022). Incidence and risk factors of COVID-19 vaccine breakthrough infections: a prospective cohort study in Belgium. Viruses.

[22] Green A, Curtis H, Hulme W, Williamson E, McDonald H, Bhaskaran K (2022). Describing the population experiencing COVID-19 vaccine breakthrough following second vaccination in England: a cohort study from OpenSAFELY. BMC Med.

[23] Kelly JD, Leonard S, Hoggatt KJ, Boscardin WJ, Lum EN, Moss-Vazquez TA (2022). Incidence of severe COVID-19 illness following vaccination and booster with BNT162b2, mRNA-1273, and Ad26.COV2.S vaccines. Jama-J Am Med Assoc..

[24] Stærke NB, Reekie J, Nielsen H, Benfield T, Wiese L, Knudsen LS (2022). Levels of SARS-CoV-2 antibodies among fully vaccinated individuals with Delta or Omicron variant breakthrough infections. Nat Commun.

[25] Haas EJ, Angulo FJ, McLaughlin JM, Anis E, Singer SR, Khan F (2021). Impact and effectiveness of mRNA BNT162b2 vaccine against SARS-CoV-2 infections and COVID-19 cases, hospitalisations, and deaths following a nationwide vaccination campaign in Israel: an observational study using national surveillance data. Lancet.

[26] Hardt K, Vandebosch A, Sadoff J, Le Gars M, Truyers C, Lowson D (2022). Efficacy, safety, and immunogenicity of a booster regimen of Ad26.COV2.S vaccine against COVID-19 (ENSEMBLE2): results of a randomised, double-blind, placebo-controlled, phase 3 trial. Lancet Infect Dis.

[27] Sadoff J, Gray G, Vandebosch A, Cárdenas V, Shukarev G, Grinsztejn B (2022). Final analysis of efficacy and safety of single-dose Ad26.COV2.S. N Engl J Med.

[28] Ella R, Reddy S, Blackwelder W, Potdar V, Yadav P, Sarangi V (2021). Efficacy, safety, and lot-to-lot immunogenicity of an inactivated SARS-CoV-2 vaccine (BBV152): interim results of a randomised, double-blind, controlled, phase 3 trial. Lancet.

[29] El SH, Baden LR, Essink B, Doblecki-Lewis S, Martin JM, Anderson EJ (2021). Efficacy of the mRNA-1273 SARS-CoV-2 vaccine at completion of blinded phase. N Engl J Med.

[30] Halperin SA, Ye L, MacKinnon-Cameron D, Smith B, Cahn PE, Ruiz-Palacios GM (2022). Final efficacy analysis, interim safety analysis, and immunogenicity of a single dose of recombinant novel coronavirus vaccine (adenovirus type 5 vector) in adults 18 years and older: an international, multicentre, randomised, double-blinded, placebo-controlled phase 3 trial. Lancet.

[31] Falsey AR, Sobieszczyk ME, Hirsch I, Sproule S, Robb ML, Corey L (2021). Phase 3 Safety and Efficacy of AZD1222 (ChAdOx1 nCoV-19) Covid-19 Vaccine. N Engl J Med.

[32] Vivaldi G, Jolliffe DA, Holt H, Tydeman F, Talaei M, Davies GA, et al. Risk factors for SARS-CoV-2 infection after primary vaccination with ChAdOx1 nCoV-19 or BNT1262b2 and after booster vaccination with BNT1262b2 or mRNA-1273: a population-based cohort study (COVIDENCE UK). 2022. 10.1016/j.lanepe.2022.100501 PMC9499825 36168404

[33] Jara A, Undurraga EA, González C, Paredes F, Fontecilla T, Jara G (2021). Effectiveness of an inactivated SARS-CoV-2 vaccine in Chile. N Engl J Med.

[34] Tan S, Pung R, Wang LF, Lye DC, Ong B, Cook AR (2022). Association of homologous and heterologous vaccine boosters with COVID-19 incidence and severity in Singapore. JAMA.

[35] Polinski JM, Weckstein AR, Batech M, Kabelac C, Kamath T, Harvey R (2022). Durability of the single-dose Ad26 COV2 S vaccine in the prevention of COVID-19 infections and hospitalizations in the US before and during the delta variant surge. JAMA Netw Open.

[36] Winkelman T, Rai NK, Bodurtha PJ, Chamberlain AM, DeSilva M, Jeruzal J (2022). Trends in COVID-19 vaccine administration and effectiveness through October 2021. JAMA Netw Open.

[37] Fabiani M, Puopolo M, Morciano C, Spuri M, Spila AS, Filia A (2022). Effectiveness of mRNA vaccines and waning of protection against SARS-CoV-2 infection and severe covid-19 during predominant circulation of the delta variant in Italy: retrospective cohort study. BMJ.

[38] Hippisley-Cox J, Coupland CA, Mehta N, Keogh RH, Diaz-Ordaz K, Khunti K (2021). Risk prediction of covid-19 related death and hospital admission in adults after covid-19 vaccination: national prospective cohort study. BMJ.

[39] Nordström P, Ballin M, Nordström A (2022). Risk of infection, hospitalisation, and death up to 9 months after a second dose of COVID-19 vaccine: a retrospective, total population cohort study in Sweden. Lancet.

[40] Katikireddi SV, Cerqueira-Silva T, Vasileiou E, Robertson C, Amele S, Pan J (2022). Two-dose ChAdOx1 nCoV-19 vaccine protection against COVID-19 hospital admissions and deaths over time: a retrospective, population-based cohort study in Scotland and Brazil. Lancet.

[41] Ford G. Prevalence vs. Incidence: what is the difference? - Students 4 Best Evidence.; 2020. Available from: https://s4be.cochrane.org/blog/2020/11/06/prevalence-vs-incidence-what-is-the-difference/. Accessed 22 Feb 2023.

[42] Liu G, Zhang B, Zhang S, Hu H, Liu T (2021). LDH, CRP and ALB predict nucleic acid turn negative within 14 days in symptomatic patients with COVID-19. Scott Med J.

[43] Altintop SE, Unalan-Altintop T, Cihangiroglu M, Onarer P, Milletli-Sezgin F, Gozukara M (2022). COVID-19 in elderly: Correlations of viral load, clinical course, laboratory parameters, among patients vaccinated with CoronaVac. Acta Microbiol Immunol Hung.

[44] Migliavaca CB, Stein C, Colpani V, Barker TH, Ziegelmann PK, Munn Z (2022). Meta-analysis of prevalence: I(2) statistic and how to deal with heterogeneity. Res Synth Methods.

[45] Bairkdar M, Rossides M, Westerlind H, Hesselstrand R, Arkema EV, Holmqvist M (2021). Incidence and prevalence of systemic sclerosis globally: a comprehensive systematic review and meta-analysis. Rheumatology (Oxford).

[46] Borenstein MHLV, Higgins JPT, Rothstein HR. Introduction to meta‐analysis. USA: Wiley, 2009. Available from: https://onlinelibrary.wiley.com/doi/book/10.1002/9780470743386. Accessed 22 Feb 2023.

[47] Willyard C (2022). What the Omicron wave is revealing about human immunity. Nature.

[48] Lian J, Yue Y, Yu W, Zhang Y (2020). Immunosenescence: a key player in cancer development. J Hematol Oncol.

[49] Moreno-Perez O, Ribes I, Boix V, Martinez-García MÁ, Otero-Rodriguez S, Reus S (2022). Hospitalized patients with breakthrough COVID-19: clinical features and poor outcome predictors. Int J Infect Dis..

[50] Díaz-Menéndez M, de la Calle-Prieto F, Montejano R, Arsuaga M, Jiménez-González M, Pérez-Blanco V (2022). Clinical characteristics and outcome of hospitalized elderly patients with COVID- 19 after vaccine failure. Vaccine.

[51] Abu-Raddad LJ, Chemaitelly H, Ayoub HH, Tang P, Coyle P, Hasan MR (2022). Relative infectiousness of SARS-CoV-2 vaccine breakthrough infections, reinfections, and primary infections. Nat Commun.

[52] Andrews N, Stowe J, Kirsebom F, Toffa S, Rickeard T, Gallagher E (2022). Covid-19 vaccine effectiveness against the Omicron (B.1.1.529) variant. N Engl J Med.

[53] Brüssow H (2021). COVID-19: vaccination problems. Environ Microbiol.

[54] COVID-19 vaccine boosters administered per 100 people. Data OWI.2023. Available from: https://ourworldindata.org/grapher/covid-vaccine-booster-doses-per-capita. Accessed 22 Feb 2023.

[55] Auster O, Finkel U, Dagan N, Barda N, Laufer A, Balicer RD (2022). Short-term adverse events after the third dose of the BNT162b2 mRNA COVID-19 vaccine in adults 60 years or older. JAMA Netw Open.

[56] Wei Z, He J, Wang C, Bao J, Leng T, Chen F (2022). The importance of booster vaccination in the context of Omicron wave. Front Immunol.

[57] Bolcato M, Rodriguez D, Feola A, Di Mizio G, Bonsignore A, Ciliberti R (2021). COVID-19 pandemic and equal access to vaccines. Vaccines (Basel).

