# Correction: SARS-CoV-2 vaccine breakthrough infection in the older adults: a meta-analysis and systematic review

**DOI:** 10.1186/s12879-023-08623-z

**Published:** 2023-10-02

**Authors:** Xiaohui Jing, Menglin Han, Xiaoxuan Wang, Li Zhou

**Affiliations:** 1 School of Chinese Materia Medica, Tianjin University of Traditional Chinese Medicine, 10 Poyang Lake Road, 301617 Tianjin, P.R. China; 2 College of Pharmaceutical Engineering of Traditional Chinese Medicine, Tianjin University of Traditional Chinese Medicine, 10 Poyang Lake Road, 301617 Tianjin, P.R. China

**BMC Infectious Diseases (2023) 23:577**

10.1186/s12879-023-08553-w

The original publication of this article contained an incorrect affiliation. The incorrect and correct information is listed in this correction article. The original article has been updated.

Incorrect:

Institute of Traditional Chinese Medicine, Tianjin University of Traditional

Chinese Medicine, 10 Poyang Lake Road, Tianjin 301617, P.R. China

Correct:

School of Chinese Materia Medica, Tianjin University of Traditional Chinese Medicine, 10 Poyang Lake Road, Tianjin 301617, P.R. China

