# Supplementary material for: SARS-CoV-2 vaccine breakthrough infection in the older adults: a meta-analysis and systematic review
Source: BMC Infect Dis. 2023 Sep 4;23:577. doi: 10.1186/s12879-023-08553-w (PMC10478381; doi:10.1186/s12879-023-08553-w)
Supplement: Supplementary file 1 — Additional file 1. [file 12879_2023_8553_MOESM1_ESM.docx]

SUPPLEMENTARY INFORMATION

Table S1. PRISMA Checklist.

| **Section and Topic** | **Item #** | **Checklist item** | **Location where item is reported** |
| --- | --- | --- | --- |
| **TITLE** | | |  |
| Title | 1 | Identify the report as a systematic review. | Title |
| **ABSTRACT** | | |  |
| Abstract | 2 | See the PRISMA 2020 for Abstracts checklist. | Abstract |
| **INTRODUCTION** | | |  |
| Rationale | 3 | Describe the rationale for the review in the context of existing knowledge. | Introduction |
| Objectives | 4 | Provide an explicit statement of the objective(s) or question(s) the review addresses. | Forth passage of the introduction |
| **METHODS** | | |  |
| Eligibility criteria | 5 | Specify the inclusion and exclusion criteria for the review and how studies were grouped for the syntheses. | Subheading 2.2 and Subheading 2.3 |
| Information sources | 6 | Specify all databases, registers, websites, organisations, reference lists and other sources searched or consulted to identify studies. Specify the date when each source was last searched or consulted. | First passage of the subheading 2.1 |
| Search strategy | 7 | Present the full search strategies for all databases, registers and websites, including any filters and limits used. | Subheading 2.1 |
| Selection process | 8 | Specify the methods used to decide whether a study met the inclusion criteria of the review, including how many reviewers screened each record and each report retrieved, whether they worked independently, and if applicable, details of automation tools used in the process. | Last paragraph of the subheading 2.1 and Figure 1. |
| Data collection process | 9 | Specify the methods used to collect data from reports, including how many reviewers collected data from each report, whether they worked independently, any processes for obtaining or confirming data from study investigators, and if applicable, details of automation tools used in the process. | Last paragraph of the subheading 2.4 |
| Data items | 10a | List and define all outcomes for which data were sought. Specify whether all results that were compatible with each outcome domain in each study were sought (e.g. for all measures, time points, analyses), and if not, the methods used to decide which results to collect. | Subheading 2.5 |
|  | 10b | List and define all other variables for which data were sought (e.g. participant and intervention characteristics, funding sources). Describe any assumptions made about any missing or unclear information. | Subheading 2.5 |
| Study risk of bias assessment | 11 | Specify the methods used to assess risk of bias in the included studies, including details of the tool(s) used, how many reviewers assessed each study and whether they worked independently, and if applicable, details of automation tools used in the process. | First paragraph of the subheading 2.4 and table 2.3.4. |
| Effect measures | 12 | Specify for each outcome the effect measure(s) (e.g. risk ratio, mean difference) used in the synthesis or presentation of results. | Subheading 2.5 |
| Synthesis methods | 13a | Describe the processes used to decide which studies were eligible for each synthesis (e.g. tabulating the study intervention characteristics and comparing against the planned groups for each synthesis (item #5)). | Figure 1 |
|  | 13b | Describe any methods required to prepare the data for presentation or synthesis, such as handling of missing summary statistics, or data conversions. | Subheading 2.5 |
|  | 13c | Describe any methods used to tabulate or visually display results of individual studies and syntheses. | Subheading 2.5 |
|  | 13d | Describe any methods used to synthesize results and provide a rationale for the choice(s). If meta-analysis was performed, describe the model(s), method(s) to identify the presence and extent of statistical heterogeneity, and software package(s) used. | Subheading 2.5 |
|  | 13e | Describe any methods used to explore possible causes of heterogeneity among study results (e.g. subgroup analysis, meta-regression). | Subheading 2.5 |
|  | 13f | Describe any sensitivity analyses conducted to assess robustness of the synthesized results. | Subheading 2.5 |
| Reporting bias assessment | 14 | Describe any methods used to assess risk of bias due to missing results in a synthesis (arising from reporting biases). | No |
| Certainty assessment | 15 | Describe any methods used to assess certainty (or confidence) in the body of evidence for an outcome. | No |
| **RESULTS** | | |  |
| Study selection | 16a | Describe the results of the search and selection process, from the number of records identified in the search to the number of studies included in the review, ideally using a flow diagram. | First paragraph of the subheading 3.1and Figure1 |
|  | 16b | Cite studies that might appear to meet the inclusion criteria, but which were excluded, and explain why they were excluded. | Subheading 2.3 and Figure1 |
| Study characteristics | 17 | Cite each included study and present its characteristics. | Subheading 2.2 and Table1 |
| Risk of bias in studies | 18 | Present assessments of risk of bias for each included study. | Table 2、Table 3 and Table 4 |
| Results of individual studies | 19 | For all outcomes, present, for each study: (a) summary statistics for each group (where appropriate) and (b) an effect estimate and its precision (e.g. confidence/credible interval), ideally using structured tables or plots. | Subheading 3.3.1 、Subheading 3.3.2、Subheading 3.4.1 and Subheading 3.4.2 Figure2、Figure3、Figure6、Figure7 |
| Results of syntheses | 20a | For each synthesis, briefly summarise the characteristics and risk of bias among contributing studies. | Table 1 and Subheading 3.2 |
|  | 20b | Present results of all statistical syntheses conducted. If meta-analysis was done, present for each the summary estimate and its precision (e.g. confidence/credible interval) and measures of statistical heterogeneity. If comparing groups, describe the direction of the effect. | Figure2、Figure3、Figure6 and Figure7 |
|  | 20c | Present results of all investigations of possible causes of heterogeneity among study results. | Subheading 3.3.1 and Figure 2  3.4.1 and Figure 6 |
|  | 20d | Present results of all sensitivity analyses conducted to assess the robustness of the synthesized results. | Figure4、Figure 5 and Figure 9 |
| Reporting biases | 21 | Present assessments of risk of bias due to missing results (arising from reporting biases) for each synthesis assessed. | table 2 table 3 table 4 |
| Certainty of evidence | 22 | Present assessments of certainty (or confidence) in the body of evidence for each outcome assessed. | No |
| **DISCUSSION** | | |  |
| Discussion | 23a | Provide a general interpretation of the results in the context of other evidence. | Forth paragraph of the discussion |
|  | 23b | Discuss any limitations of the evidence included in the review. | Study limitations |
|  | 23c | Discuss any limitations of the review processes used. | No |
|  | 23d | Discuss implications of the results for practice, policy, and future research. | Publication implications |
| **OTHER INFORMATION** | | |  |
| Registration and protocol | 24a | Provide registration information for the review, including register name and registration number, or state that the review was not registered. | No |
|  | 24b | Indicate where the review protocol can be accessed, or state that a protocol was not prepared. | No |
|  | 24c | Describe and explain any amendments to information provided at registration or in the protocol. | No |
| Support | 25 | Describe sources of financial or non-financial support for the review, and the role of the funders or sponsors in the review. | Subheading Funding |
| Competing interests | 26 | Declare any competing interests of review authors. | Subheading Conflicts of Interest |
| Availability of data, code and other materials | 27 | Report which of the following are publicly available and where they can be found: template data collection forms; data extracted from included studies; data used for all analyses; analytic code; any other materials used in the review. | Subheading Data Availability Statement |

Supplement 1

Search Strategy of Pubmed

(1) “COVID-19 breakthrough infections” [Title/Abstract]) OR “breakthrough COVID-19 infections” [Title/Abstract] OR “COVID-19 breakthrough infection” [Title/Abstract] OR “breakthrough infection COVID-19” [Title/Abstract])) OR “breakthrough COVID-19 infection” [Title/Abstract] OR “breakthrough infections COVID-19” [Title/Abstract] OR “COVID-19 breakthrough” [Title/Abstract]

(2) “elderly” [Title/Abstract] OR “aged” [Title/Abstract]))

(3)“COVID 19 Vaccines”[Title/Abstract] OR “Vaccines, COVID-19”[Title/Abstract] OR “COVID-19 Virus Vaccines”[Title/Abstract] OR“COVID 19 Virus Vaccines”[Title/Abstract] OR “Vaccines, COVID-19 Virus”[Title/Abstract] OR “Virus Vaccines, COVID-19”[Title/Abstract] OR “COVID-19 Virus Vaccine”[Title/Abstract] OR “COVID 19 Virus Vaccine”[Title/Abstract] OR “Vaccine, COVID-19 Virus”[Title/Abstract] OR “Virus Vaccine, COVID-19”[Title/Abstract] OR “COVID19 Virus Vaccines”[Title/Abstract] OR “Vaccines, COVID19 Virus”[Title/Abstract] OR “Virus Vaccines, COVID19”[Title/Abstract] OR “COVID19 Virus Vaccine”[Title/Abstract] OR “Vaccine, COVID19 Virus”[Title/Abstract] OR “Virus Vaccine, COVID19”[Title/Abstract] OR “COVID19 Vaccines”[Title/Abstract] OR “Vaccines, COVID19”[Title/Abstract] OR “COVID19 Vaccine”[Title/Abstract] OR “Vaccine, COVID19”[Title/Abstract] OR “SARS-CoV-2 Vaccines”[Title/Abstract] OR “SARS CoV 2 Vaccines”[Title/Abstract] OR “Vaccines, SARS-CoV-2”[Title/Abstract] OR “SARS-CoV-2 Vaccine”[Title/Abstract] OR “SARS CoV 2 Vaccine”[Title/Abstract] OR “Vaccine, SARS-CoV-2”[Title/Abstract] OR “SARS2 Vaccines”[Title/Abstract] OR “Vaccines, SARS2”[Title/Abstract] OR “SARS2 Vaccine”[Title/Abstract] OR “Vaccine, SARS2”[Title/Abstract] OR “Coronavirus Disease 2019 Vaccines”[Title/Abstract] OR “Coronavirus Disease 2019 Vaccine”[Title/Abstract] OR “Coronavirus Disease 2019 Virus Vaccine”[Title/Abstract] OR “Coronavirus Disease 2019 Virus Vaccines”[Title/Abstract] OR “Coronavirus Disease-19 Vaccines”[Title/Abstract] OR “Coronavirus Disease 19 Vaccines”[Title/Abstract] OR “Vaccines, Coronavirus Disease-19”[Title/Abstract] OR “Coronavirus Disease-19 Vaccine”[Title/Abstract] OR “Coronavirus Disease 19 Vaccine”[Title/Abstract] OR “Vaccine, Coronavirus Disease-19”[Title/Abstract] OR “COVID 19 Vaccine”[Title/Abstract] OR “Vaccine, COVID 19”[Title/Abstract] OR “2019-nCoV Vaccine”[Title/Abstract] OR “2019 nCoV Vaccine”[Title/Abstract] OR “Vaccine, 2019-nCoV”[Title/Abstract] OR “2019 Novel Coronavirus Vaccines”[Title/Abstract] OR “2019 Novel Coronavirus Vaccine”[Title/Abstract] OR “2019-nCoV Vaccines”[Title/Abstract] OR “2019 nCoV Vaccines”[Title/Abstract] OR “Vaccines, 2019-nCoV”[Title/Abstract] OR “COVID-19 Vaccine”[Title/Abstract] OR “Vaccine, COVID-19”[Title/Abstract] OR “SARS Coronavirus 2 Vaccines”[Title/Abstract]

And (4) #1 and #2 and #3

Search Strategy of Embase

(1) ('aged'/exp OR 'frail elderly'/exp OR 'aging'/exp OR 'gerontopsychiatry'/exp OR 'older*' OR 'older adult*' OR 'olds' OR 'elder*' OR 'aging' OR 'ageing' OR 'aged' OR 'senior*' OR 'geriatr*' OR 'gerontolog*' OR 'late-life' OR 'late life' OR 'vulnerab*')

(2) ('vaccine'/exp OR 'SARS-CoV-2 vaccine'/exp OR 'vaccination'/exp OR 'vaccin*':ab,ti OR 'immunis*':ab,ti OR 'immuniz*':ab,ti)

(3) ('break-through infection'/exp OR 'break-through infection')

(4) # 1 AND # 2 AND # 3

Search Strategy of Web of Science

(ALL=("Aged" OR "Aged, 80 and over" OR "Frail Elderly" OR "older*" OR "older adult*" OR "olds" OR "elder*" OR "aging" OR "ageing" OR "senior*" OR "geriatr*" OR "gerontolog*" OR "late-life" OR "late life" OR "vulnerab*")) AND ((((((ALL=(COVID-19 breakthrough infections)) OR ALL=(breakthrough COVID-19 infections)) OR ALL=(COVID-19 breakthrough infection)) OR ALL=(breakthrough infection COVID-19)) OR ALL=(breakthrough COVID-19 infection)) OR ALL=(breakthrough infections COVID-19)) OR ALL=(COVID-19 breakthrough) AND (TS=("vaccin*" OR "immunis*" OR "immuniz*"))

Search Strategy of Cochrane Library

# 1 (SARS-Cov-2 Breakthrough infection):ti,ab,kw

(Word variations have been searched)

# 2 MeSH descriptor: [COVID-19 Vaccines] explode all trees.

# 3 MeSH descriptor: [Vaccines] in all MeSH products.

# 4 vaccin* or immunis* or immuniz*

# 5 # 2 or # 3 or # 4.

# 6 MeSH descriptor: [Aged] in all MeSH products.

# 7 MeSH descriptor: [Aged, 80 and over] explode all trees.

# 8 MeSH descriptor: [Frail Elderly] explode all trees.

# 9 older* or “older adult” or olds or elder* or aging or ageing or aged or ag*ing or seni* or geriatr* or gerontolog* or “late-life” or “late life” or vulnerab*

# 10 # 6 or # 7 or # 8 or # 9

# 11 # 1 and # 5 and # 10
